# Supplementary material for: Inferring multilayer interactome networks shaping phenotypic plasticity and evolution
Source: Nat Commun. 2021 Sep 6;12:5304. doi: 10.1038/s41467-021-25086-5 (PMC8421358; doi:10.1038/s41467-021-25086-5)
Supplement: Supplementary file 1 — Supplementary Figures [file 41467_2021_25086_MOESM1_ESM.pdf]

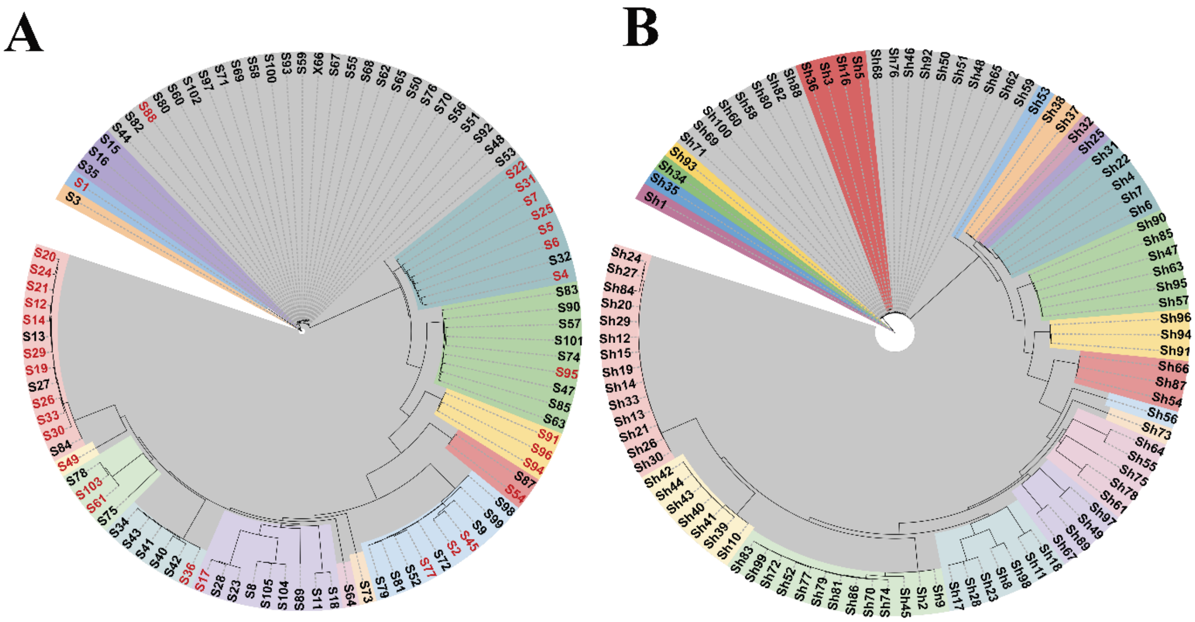

Figure S1 Phylogenetic trees of *S. aureus* strains used in two independent GWAS experiments of phenotypic plasticity. (**A**) The abiotic GWAS experiment includes 99 strains, labeled as S1 – S9, S11 – 42, S47 – S105. Most of the strains that cannot grow in 6 μg/mL vancomycin-exposed media (highlighted in red) tend to be from particular phylogenetic groups. (**B**) The biotic GWAS experiment includes 100 strains, labeled by Sh1 – Sh100. Phylogenetic groups are broadly consistent with subpopulations detected.

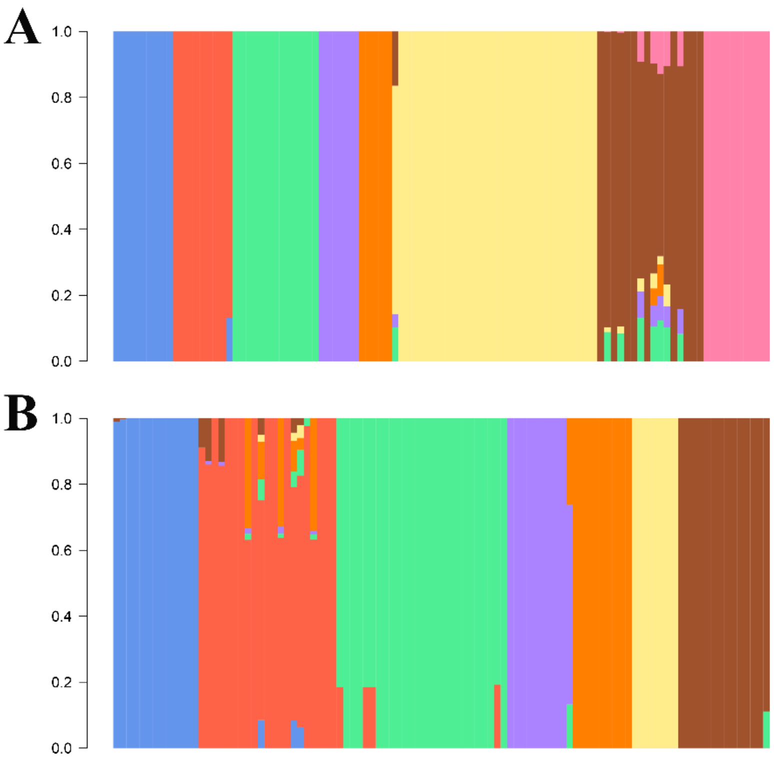

Figure S2 The detection of subpopulations (delimited by a different color) among a different panel of *S. aureus* strains used in two independent GWAS experiments of abiotic (**A**) and biotic phenotypic plasticity (**B**).

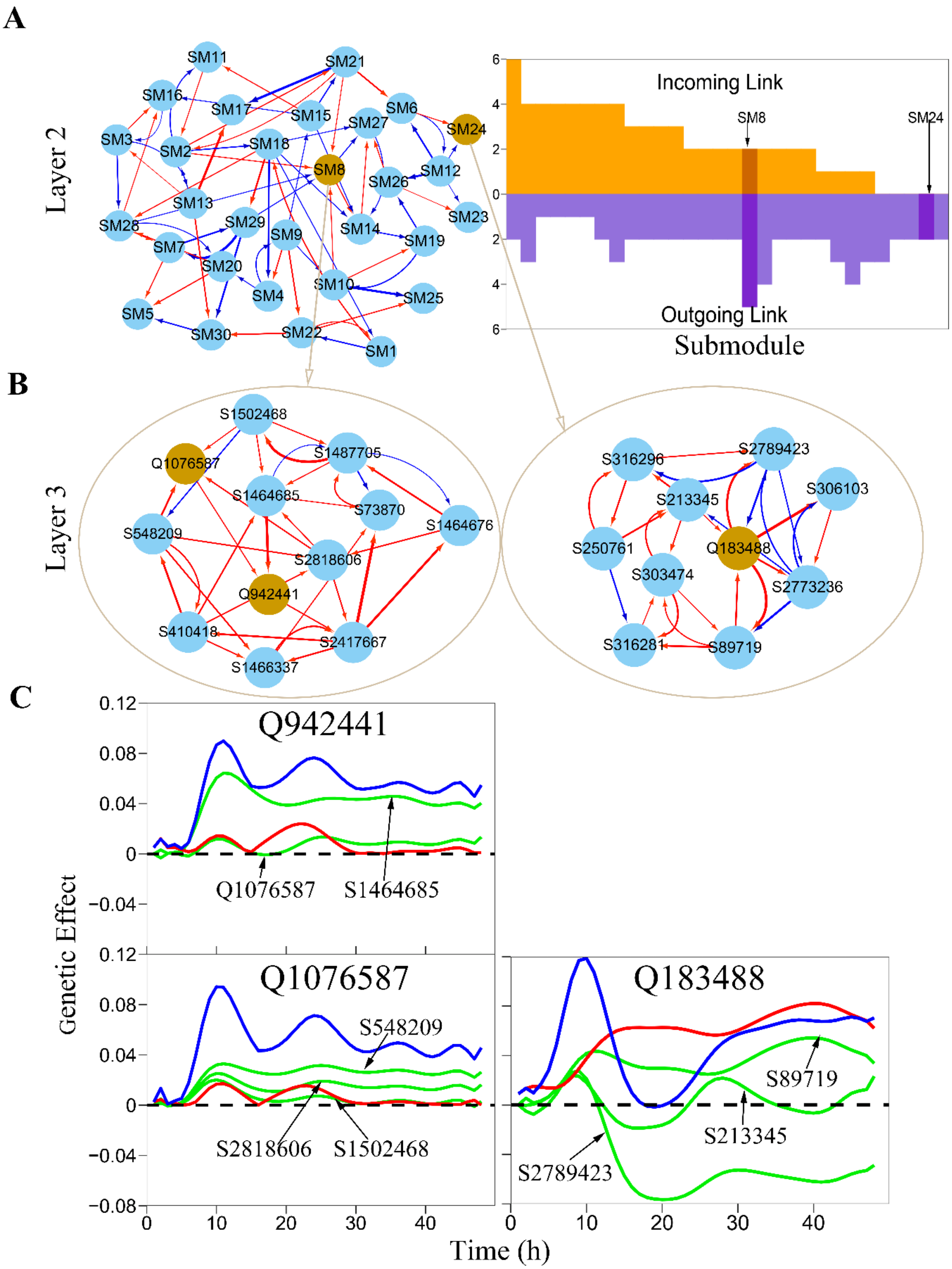

Figure S3 The second- and third-layer networks of QTL-containing module M10. (**A**) Left panel: The 30-node inter-submodule network at the second layer under network community M10 of the first-layer network. Submodules SM8 and SM24 contain QTLs, highlighted in orange. Red and blue arrowed lines stand for the direction of up-regulation and down-regulation, respectively,

with the thickness of lines proportional to the strength of regulation. Right panel: Distribution of the number of outgoing and incoming links over 30 submodules. The positions of QTL-containing submodules are indicated. (**B**) The 12-node and 10-node inter-SNP network at the third later under QTL-containing network subcommunities SM8 and SM24, respectively, of the second-layer network M10. QTLs are highlighted in orange. (**C**) Curves of genetic effects on phenotypic plasticity for QTLs from SM8 and SM24. Net genetic effect (blue line) is decomposed into independent effect (red line) and dependent effects (green line) due to regulation by other SNPs (regulators' names are indicated).

.

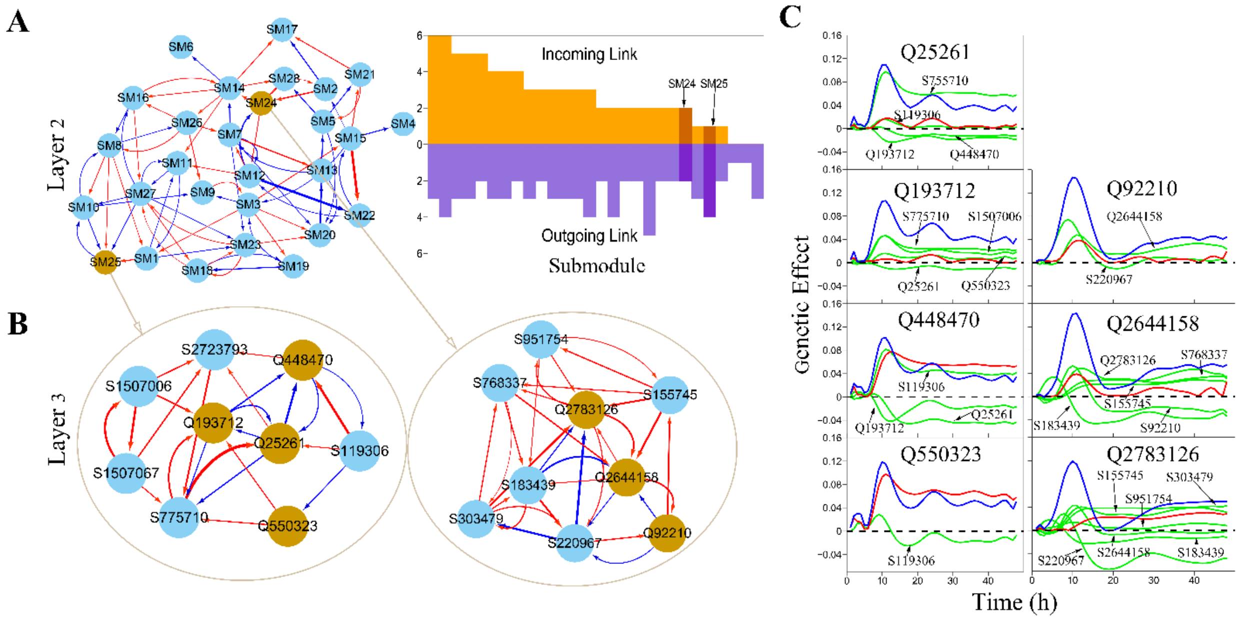

Figure S4 The second- and third-layer networks of QTL-containing module M7. (**A**) Left panel: The 27-node inter-submodule network at the second layer under network community M7 of the first-layer network. Submodules SM24 and SM25 contain QTLs, highlighted in orange. Red and blue arrowed lines stand for the direction of up-regulation and down-regulation, respectively, with the thickness of lines proportional to the strength of regulation. Right panel: Distribution of the number of outgoing and incoming links over 27 submodules. The positions of QTL-containing submodules are indicated. (**B**) The 9-node, and 9-node inter-SNP network at the third later under QTL-containing network subcommunities SM24 and SM25, respectively, of the second-layer network M7. QTLs are highlighted in orange. (**C**) Curves of genetic effects on phenotypic plasticity for QTLs from SM24 and SM25. Net genetic effect (blue line) is decomposed into independent effect (red line) and dependent effects (green line) due to regulation by other SNPs (regulators' names are indicated).

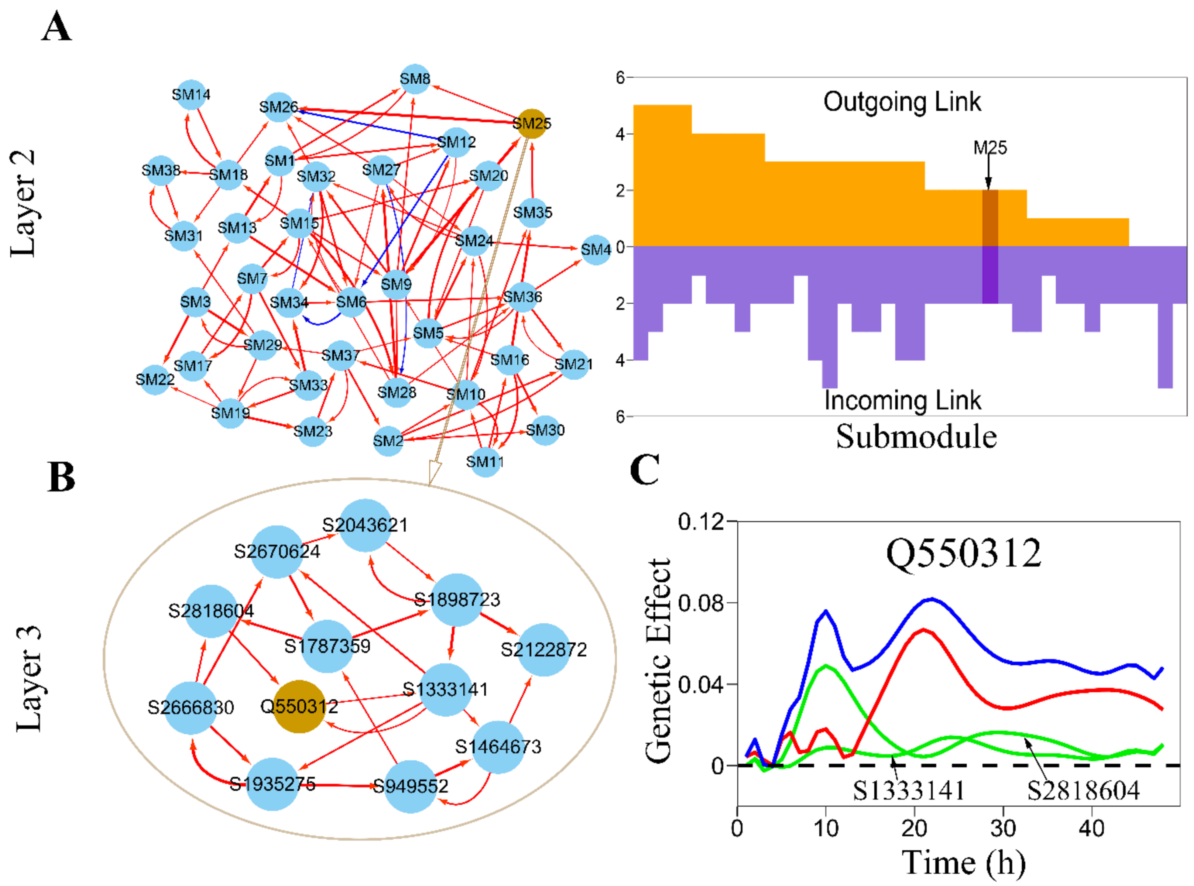

Figure S5 The second- and third-layer networks of QTL-containing module M14. (**A**) Left panel: The 38-node inter-submodule network at the second layer under network community M14 of the first-layer network. Submodule SM25 contains QTLs, highlighted in orange. Red and blue arrowed lines stand for the direction of up-regulation and down-regulation, respectively, with the thickness of lines proportional to the strength of regulation. Right panel: Distribution of the number of outgoing and incoming links over 38 submodules. The position of the QTL-containing submodule is indicated. (**B**) The 12-node inter-SNP network at the third later under QTL-containing network subcommunities SM25 of the second-layer network M14. A QTL is highlighted in orange. (**C**) Curves of genetic effects on phenotypic plasticity for the QTL from SM25. Net genetic effect (blue line) is decomposed into independent effect (red line) and dependent effects (green line) due to regulation by other SNPs (regulators' names are indicated).

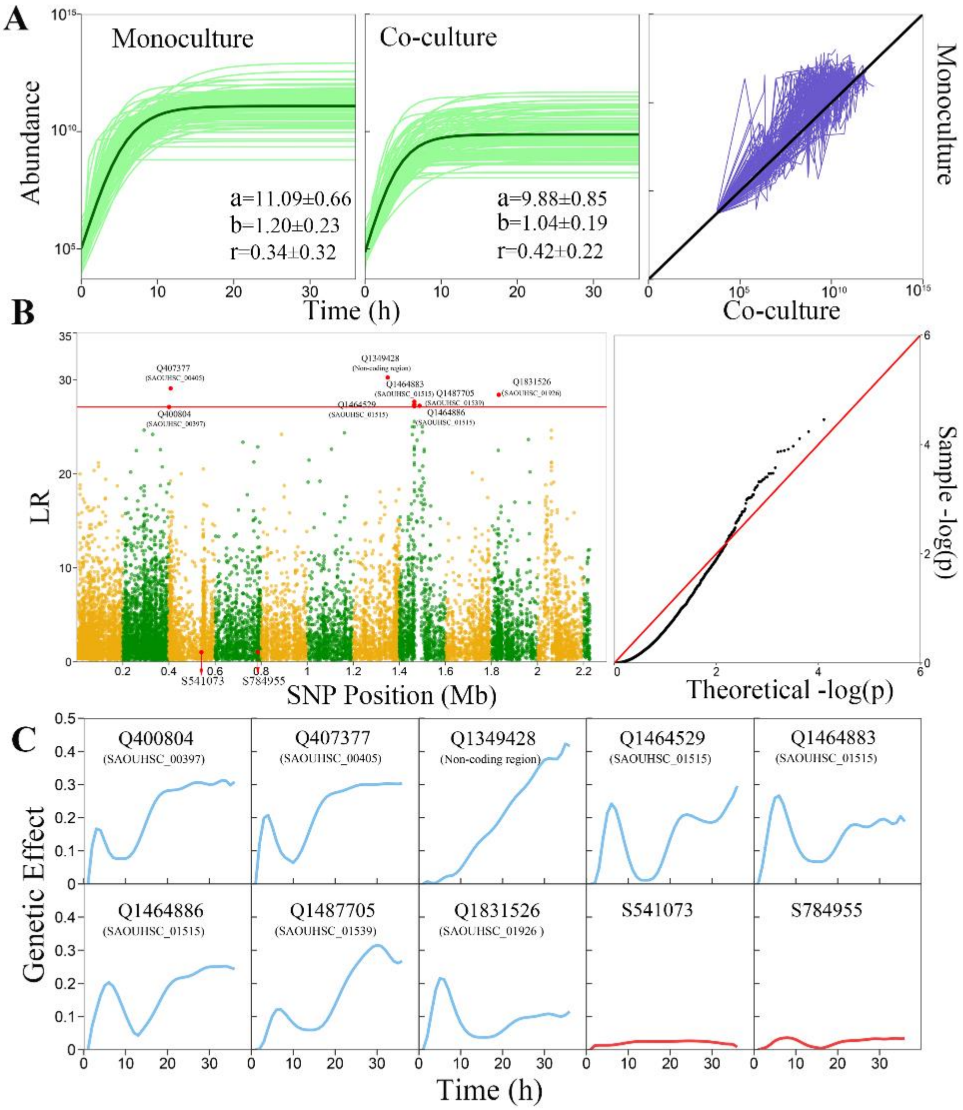

Figure S6 A general flowchart of genetic mapping for dynamic complex traits. (**A**) Growth trajectories of 99 *S. aureus* strains (grey think lines) cultured in vancomycin-free (control, 0 μg/mL) and vancomycin-stress (6 μg/mL) media. The mean growth is fitted by a logistic equation (think green line), from which the timing of maximum growth rate t_I is estimated. The square plot of strain growth in control against stress is shown, where the degree of deviation from the diagonal line is positively associated with vancomycin-induced difference in growth curve. (**B**) Left panel: Manhattan plot of the significance test based on log-likelihood ratio (LR) for associations between the phenotypic plasticity of microbial growth trajectory and SNPs distributed on the *S. aureus* genome. coFunMap identifies 16 significant SNPs (called QTLs) that are indicated above red horizontal line representing the genome-wide critical threshold determined from 1000 permutation tests. At the bottom of the Manhattan plot, five insignificant SNPs that are chosen for subsequent dissection are indicated. Right panel: Q-Q plot used to characterize the extent of deviation of the observed distribution of the test statistic from the expected (null) distribution for phenotypic plasticity-related GWAS of 99 *S. aureus* strains. (**C**) Curves of genetic effects on phenotypic plasticity for 16 QTLs and five chosen insignificant SNPs estimated by coFunMap.

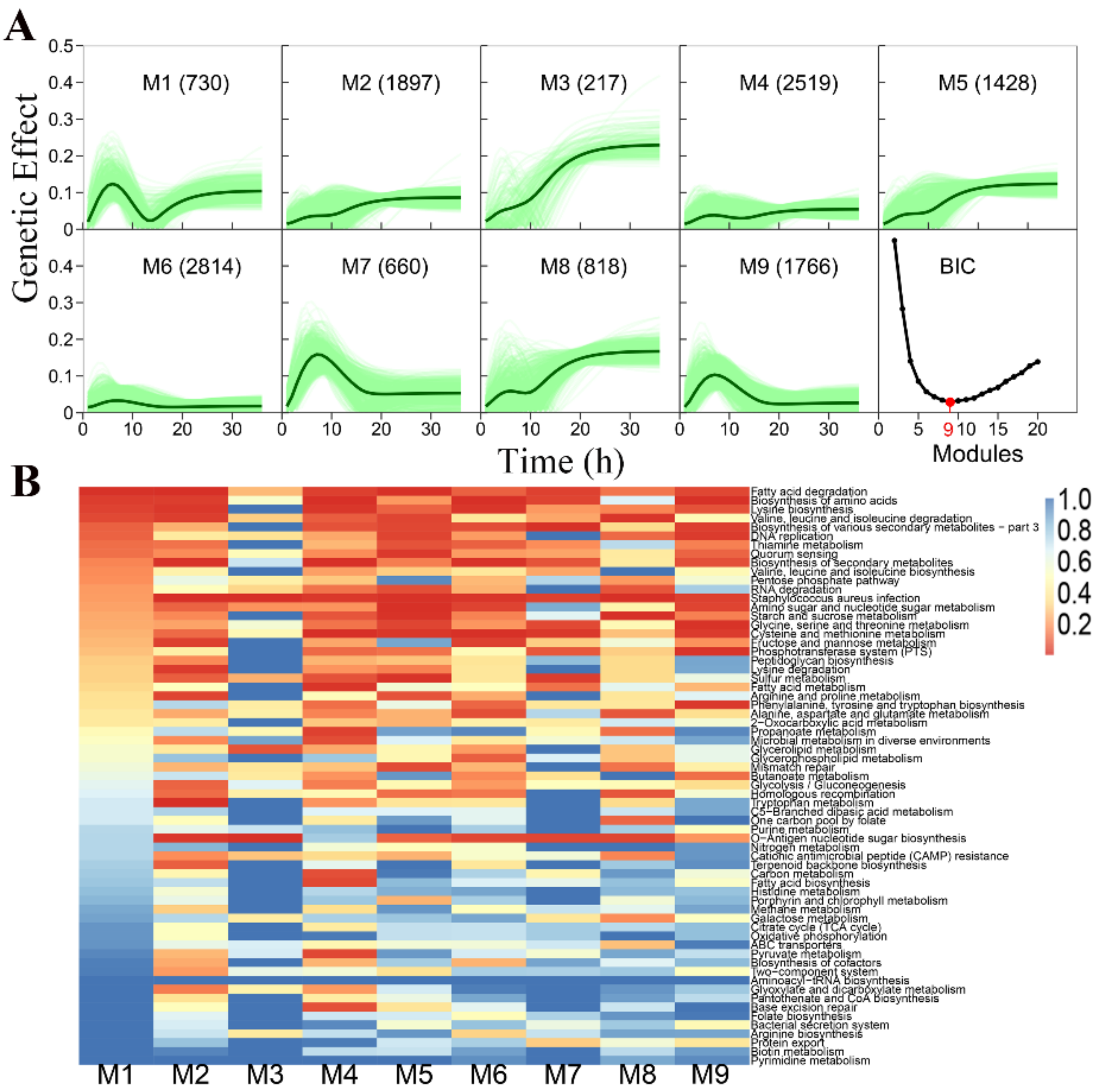

Figure S7 (**A**) The curves of genetic effects on the biotic phenotypic plasticity of microbial growth trajectory for nine distinct modules (the number of SNPs within each module given) determined according to BIC. Thick green line represents the mean curve of all SNPs within modules, with individual SNP-specific curves denoted by thin green lines. (**B**) Heatmap of gene functions from nine modules by gene set enrichment analysis.

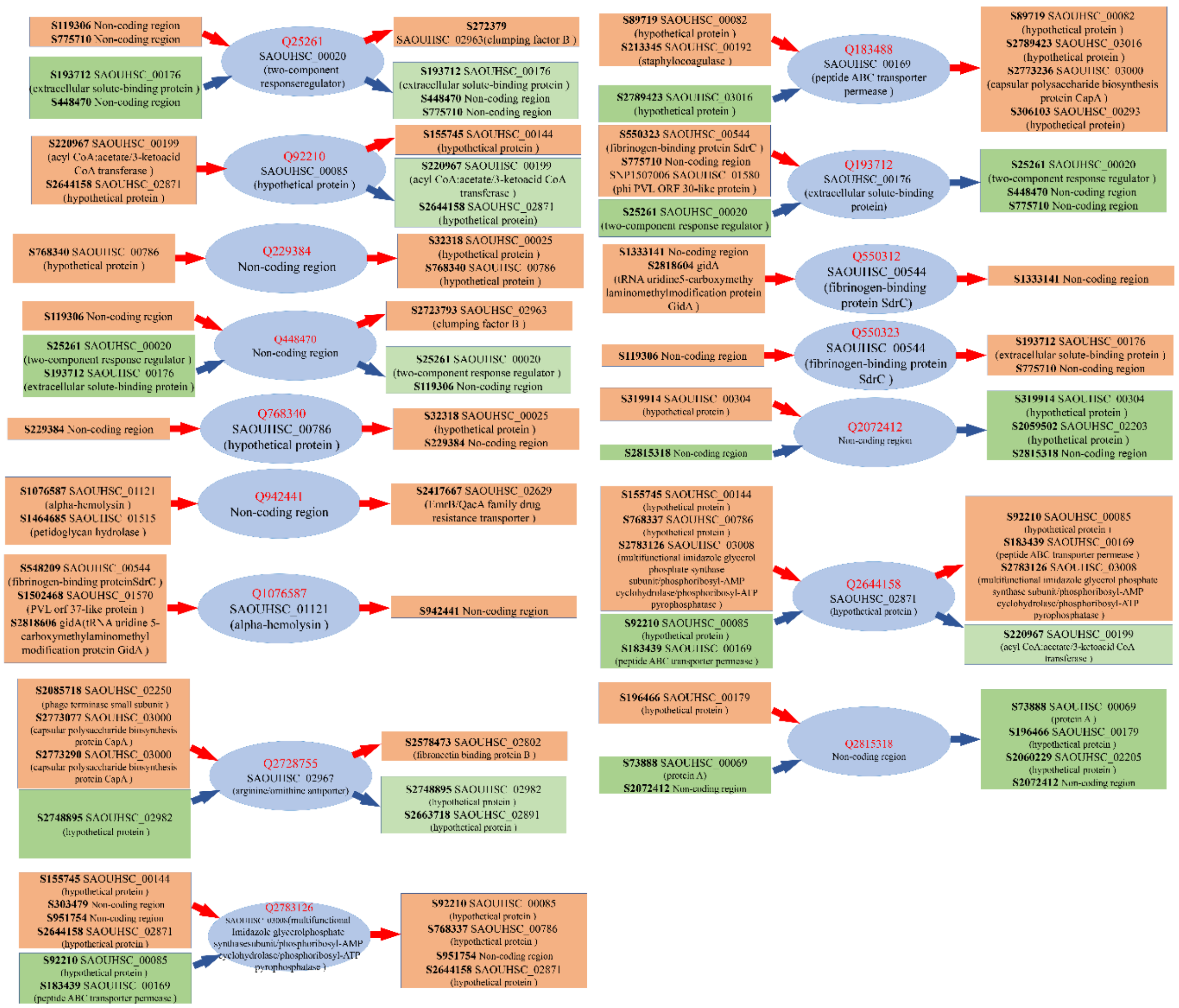

Figure S8 Gene enrichment analysis of QTLs for the phenotypic plasticity of microbial growth trajectory to vancomycin stress detected by coFunMap and the SNPs that regulate the plasticity QTLs. A QTL is up-regulated (red arrows) by SNPs within warm frames and/or down-regulated (blue arrows) by SNPs within cold frames.

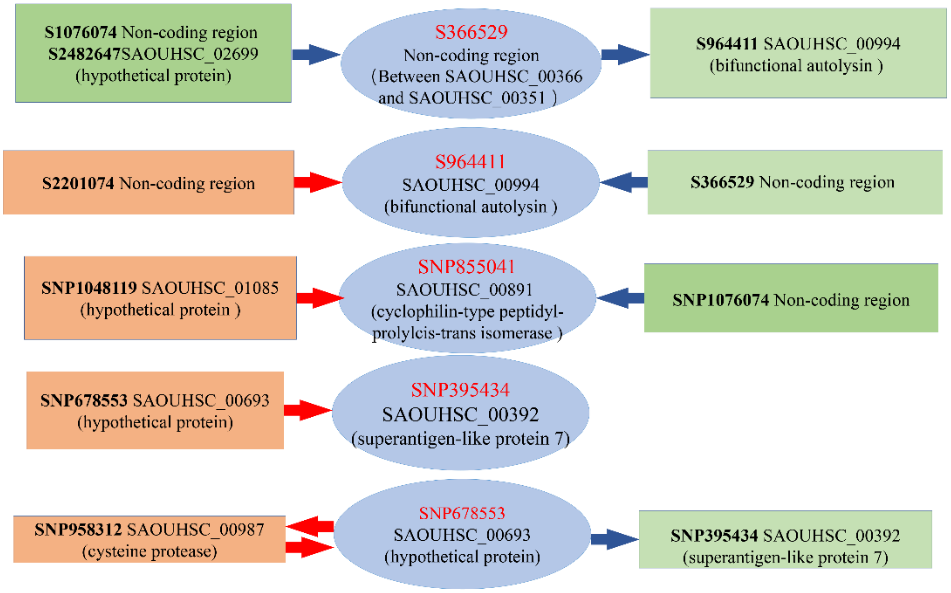

Figure S9 Gene enrichment analysis of five insignificant SNPs (shown in the ovals of the middle panel) for the phenotypic plasticity of microbial growth trajectory according to coFunMap and all those SNPs that interact with the former in response to vancomycin stress. An insignificant SNP is up-regulated (red arrows) by SNPs within warm frames and down-regulated (blue arrows) by SNPs within cold frames. The pattern and strength of regulation for each SNP by other loci may be an important but neglected driver of phenotypic plasticity.
