# Supplementary material for: Inferring multilayer interactome networks shaping phenotypic plasticity and evolution
Source: Nat Commun. 2021 Sep 6;12:5304. doi: 10.1038/s41467-021-25086-5 (PMC8421358; doi:10.1038/s41467-021-25086-5)
Supplement: Supplementary file 4 — List of Supplementary Data files [file 41467_2021_25086_MOESM4_ESM.pdf]

Data set 1: Background of 99 S. aureus strains used in the abiotic GWAS experiment.

Data set 2: Gene enrichment analysis of QTLs for abiotic phenotypic plasticity of S. aureus to vancomycin exposition detected by coFunMap.

Data set 3: Gene enrichment analysis of QTLs for biotic phenotypic plasticity of S. aureus to E. coli coexistence detected by coFunMap.

Data set 4: Resequencing statistics of S. aureus strains used in the biotic GWAS experiment.
