# Supplementary material for: Inferring multilayer interactome networks shaping phenotypic plasticity and evolution
Source: Nat Commun. 2021 Sep 6;12:5304. doi: 10.1038/s41467-021-25086-5 (PMC8421358; doi:10.1038/s41467-021-25086-5)
Supplement: Supplementary file 3 — Editorial Assessment Report [file 41467_2021_25086_MOESM3_ESM.pdf]

nature portfolio
GUIDED OPEN ACCESS
nature genetics nature communications communications biology

Editorial Assessment Report

Dear Professor Wu,

Thank you again for choosing to submit your manuscript using the Guided Open Access pilot at the Nature Portfolio. As part of this process, our editorial team has considered your paper for three of our journals with strong interest in publishing in your field: *Nature Genetics*, *Nature Communications*, and *Communications Biology*.

Your manuscript entitled "Inferring multilayer interactome networks shaping phenotypic plasticity and evolution" has now been reviewed by 4 experts in biostatistics, phenotypic plasticity, and bacterial genomics, whose comments are included in the attached Editorial Assessment Report. As part of the Guided Open Access pilot, editors from all 3 journals have discussed the reviewer reports and the manuscript's suitability for our journals. After careful evaluation, our editorial recommendation is to revise the manuscript and submit back through the Guided Open Access submission portal for consideration at *Communications Biology* or *Nature Communications*. Provided the revisions satisfy all technical and editorial concerns, *Communications Biology* is especially interested in publishing your manuscript. Please see details in the attached Editorial Assessment Report.

In brief, for publication in *Communications Biology*, we would require you to address the referee concerns regarding methodological advance and benchmarking over related tools, pathway analyses, and limited growth curve or MIC data. Furthermore, all code should be made immediately available for reviewer input. For consideration in *Nature Communications*, the editors would request all of these edits, as well as additional demonstration of utility in at least one separate dataset. You will find more details regarding the required revisions in the Editorial Assessment Report below.

Please note that the Editorial Assessment Report is a standalone document that contains an editorial evaluation, recommendation and portable peer advice to help you navigate and interpret the reviewers' reports. It also provides guidance for adhering to best practice with regard to transparency and reproducibility, for example on the issue of sharing data. We have also included information about data accessibility and reproducibility, which we hope you find useful.

Should you have any questions about the recommended journals or would like advice on the revisions, you can contact me directly and I will be happy to assist. We look forward to receiving the revised version of your manuscript.

Yours sincerely,
George Inglis
--
George Inglis
Associate Editor
Communications Biology

On behalf of the Guided OA editorial team

Contents of this Report

- **Manuscript overview**: details about your manuscript and the editorial team.
- **Manuscript assessment**: personalised recommendation from the editors.
- **About the editorial process**: an overview of the Guided Open Access process.
- **Annotated reviewer comments**: the referee reports with comments from the editors.
- **Open research evaluation**: advice for adhering to best reproducibility practices.

Manuscript overview

Manuscript number	Submission date	Decision date
GUIDEDOA-21-00023	30 January 2021	4 March 2021

**Title**	Inferring multilayer interactome networks shaping phenotypic plasticity and evolution	**Corresponding author**	Rongling Wu<br>ORCID: 0000-0002-2334-6421
**Preprint information**	No preprints found.	**Peer review type**	Single-blind
**Editorial Assessment Team**	**Primary editor: George Inglis**<br>**Home Journal:** *Communications Biology*, ORCID: 0000-0002-9069-5242<br>**Editorial team members:**<br>Wei Li, *Nature Genetics*, ORCID: 0000-0002-7885-1775<br>Rebecca Furlong, *Nature Communications*, ORCID: 0000-0002-8451-6471		
**About your primary editor**	George received his PhD in Genetics and Molecular Biology from Emory University, where he studied mouse models of voltage-gated sodium channel dysfunction and epilepsy. He also has research experience in epigenomics and in vitro models of neuronal development. George joined the editorial team of *Communications Biology* in September 2020 and is based in the New York office.		

Manuscript assessment and recommendation

**Editor's summary of the manuscript**	Here the authors present a computational model to infer omnigenic interactome networks underlying phenotypic plasticity. By incorporating developmental modularity theory and high-dimensional statistical models, the new framework can reconstruct multilayer large-scale genetic networks from any number of SNPs.<br><br>One benefit of this study is that is one of the first applications of omnigenic theory toward a bacterial system. However, several referees commented on unclear rationale behind the computational model, and whether it presented a substantial advance over previous applications of bacterial GWAS or QTL analysis. There were also concerns about the applicability of this approach to other datasets or traits. While we would require the code to be made publicly available at the time of publication, we believe it is critical for the code and input genomic data to be provided to reviewers.<br><br>In summary, a resubmission should include, at a minimum, the following revisions:<br><br>(1) Benchmarking the predictive ability of the omnigenic model to other tools, as suggested by Referee #4<br><br>(2) Provide all code and data accessions, as best highlighted by Referee #1, and greatly elaborate on how the model works in the Methods. It would also be useful to include tables of required inputs and a broader discussion of any advantages/limitations, as suggested by Referee #2.<br><br>(3) The Introduction and Discussion should be updated with context about current knowledge on vancomycin resistance (per Referee #1), how their model varies from normal adaptive evolution (Referee #3), and the underlying rationale for the study (Referee #4).<br><br>(4) Gene modules should be updated with pathway analyses to infer biological context, and more MIC or growth curve data should be provided, as suggested by Referee #1.

Editorial assessment overview

Journal	Assessment	Details
Nature Genetics	**Not feasible to meet the editorial requirements**	The technical advance (compared to prior literature) represented by the method is not sufficient for publication in *Nature Genetics*.
Nature Communications	**Major revisions needed**	All revisions requested by *Communications Biology* (see below), plus analysis in an additional dataset would be required for publication.
Communications Biology	**Major revisions needed**	As commented by the reviewers, improved reporting and benchmarking, pathway analysis, and MIC/growth curve data are required for publication.

**Editor's recommendation**	***Option 1*: Revise for consideration at Communications Biology**<br>While many revisions are needed, these do not require any new datasets or expansion of the work beyond proper benchmarking of the predictive ability of the approach compared to other tools. See the annotated reviewer comments for details.<br><br>***Option 2*: Revise for consideration at Nature Communications**<br>For publication in *Nature Communications*, the authors would need to demonstrate more general applicability, by including analysis of another dataset. The editors would also need to be convinced of the broad utility of the method and its advantages over existing tools.<br><br>**Please state in your cover letter which journal you have revised for.**
**Next steps**	If you would like to follow our recommendation, when you are ready you can upload the revised manuscript, along with your point-by-point response to the reviewer's reports and editorial advice **here*.** If you need assistance with our manuscript tracking system, please contact Adam Lipkin, our Nature Portfolio Guided OA support specialist at guidedOA@nature.com.<br>**This URL links to your confidential home page and associated information about manuscripts you may have submitted, or that you are reviewing for us.*

About the editorial process

By selecting the Nature Portfolio Guided Open Access option, your manuscript was assessed for suitability in three of our titles that provide venues for publication of high-quality work across the spectrum of genetics research: *Nature Genetics, Nature Communications*, and *Communications Biology*. For more information about Guided Open Access, please see here.

Collaborative editorial assessment

Your editorial team discussed the manuscript to determine its suitability for the Nature Portfolio Guided OA pilot. Our assessment of your manuscript takes into account several factors, including whether the work meets the **technical standard** of the Nature Portfolio and whether the findings are of **immediate significance** to the readership of at least one of the participating journals in the Nature Portfolio Guided Open Access genetics cluster.

Peer review

Experts were asked to evaluate the following aspects of your manuscript:

Novelty in comparison to prior publications;

Likely audience of researchers in terms of broad fields of study and size;

Potential impact of the study on the immediate or wider research field;

Evidence for the claims and whether additional experiments or analyses could feasibly strengthen the evidence;

Methodological detail and whether the manuscript is reproducible as written;

Appropriateness of the literature review.

Editorial evaluation of reviews

Your editorial team discussed the potential suitability of your manuscript for each of the participating journals. They then discussed the revisions necessary in order for the work to be published, keeping each journal's specific editorial criteria in mind.

Journals in the Nature portfolio will support authors wishing to transfer their reviews and (where reviewers agree) the reviewers' identities to journals outside of Springer Nature. For any questions about review portability, please contact our editorial office: guidedoa@nature.com

Annotated Reviewer Reports

The editor has included some additional comments on the specific points raised by the reviewers below. However, please note that all points should be addressed in a revision, even if the editor has not specifically commented on them.

**Reviewer #1**<br>*Report received: 25 February 2021*	
**Reviewer #1**	This reviewer has not chosen to waive anonymity. The reviewer's identity can only be shared with representatives of an established journal editorial office.
**Reviewer #1 expertise**<br>*Summarised by the editor*	This reviewer has expertise in computational biology and bacterial GWAS.
**Editor's comments about this review**	All points raised by this reviewer regarding data and code availability should be addressed in a revision, prior to consideration by *Communications Biology* or *Nature Communications*. Reviewer #1 also raises excellent points about MIC or growth curves (comments #5/7) and pathway analyses (comment #10) that should be addressed.
**Reviewer #1 comments**	
**Overview**	The approach authors have taken to map the genetic effects, and relationships between them, on a bacterial phenotype is novel and interesting. The authors do a good job at citing previous methodological papers, but failed to cite relevant literature on the genetic basis of vancomycin non-susceptibility in *Staphylococcus aureus*, which is needed to contextualise and assess the biological plausibility of their findings.<br><br>In this study, Yang and colleagues study the genetic basis of differences in bacterial growth between two different experimental/environmental conditions, what they term 'phenotypic plasticity'. Specifically, they measure growth differences over time of *Staphylococcus aureus* strains exposed to vancomycin vs. not exposed in vitro. They next try to calculate the genetic effects of QTL, gene network modules and individual SNPs on growth differences.<br><br>The current main limitations of the work are (1) the over-use of technical terms which makes it difficult to read and (2) the lack of biological interpretation and contextualisation of the findings.<br><br>Specifically, given the large scientific literature on the genetic basis of vancomycin non-susceptibility in *S. aureus*, a comparison of the discovered QTLs, and biological functions of gene modules, with known vancomycin-resistance-conferring genes is

	currently missing. See individual comments below:

Specific comments

#	Reviewer comment	Editorial comment
**1**	Lines 794-800. Avoid repetition with introduction on the clinical relevance of *S. aureus* and vancomycin.	
**Methods and data availability**		
**2**	Details on bioinformatic pipelines to process bacterial genomic data, including SNP calling, is currently missing. Detailed information on software tools, versions and command lines used are needed.	
**3**	Although the 'Data availability' statement indicates that 'All data and code used in the manuscript are publicly available', no link to a GitHub page or to other code repositories could be find in the manuscript, so it is not clear where the code can be found.	
**4**	URL links to the repositories storing the genomic data should also be included. The 'serial number' [better named as 'accession number'] in Table S2 allowed me to find the genomic data on https://bigd.big.ac.cn/bioproject/browse/PRJCA001035 and https://bigd.big.ac.cn/bioproject/browse/PRJCA002084. The authors should make sure the genomic data is also available on either the SRA NCBI or ENA EBI, as stablished software tools exist to retrieve genomic data from these widely used repositories.	**Please make genomic data immediately available for reviewers, though we would recommend using a separate repository as described in the Open Research evaluation below (complete data policy here). Genomic data would ultimately have to be deposited prior to acceptance for both *Communications Biology* and *Nature Communications*.**

**5**	Lines 812-827. The authors only made one MIC measurement available per strain in Table S2, while they reported more than one, specifically: (1) the initial MIC measurement of the parental strain (is this the one presented in Table S2?), (2) MICs after 60 days of in vitro treatment and (3) measurements of abundance [in what units?] at 14 time points "under two treatments, one containing 6 µg/mL vancomycin and the other being vancomycin-free (control)", that is, the source data of Figure 1A. All measurements must be made available to ensure reproducibility. The same applies for the growth measurements taken at 4µg/mL and 2µg/mL concentrations of vancomycin.	
**Results**		
**6**	Figure 1B. The term "phenotypic plasticity of microbial growth trajectory" described as the phenotype used in the GWAS may not easy to interpret for the general reader. Please state in more plain terms what measurement was used as the outcome/phenotype variable in the GWAS, for example, "difference of microbial abundance between control and stress conditions", or, as stated in the discussion "difference of growth trajectory" between conditions. Also, coFunMap, the method used to map QTLs, is not described in the Methods, please add citation to the method/tool and details of parameters used. It is also important that QTLs are annotated on the Manhattan plot with their gene name and nucleotide or amino acid change, so that specialised readers can recognise genes.	
**7**	Lines 114-125. The authors note that strains differ notably in the way they grow under vancomycin stress, compared to how the grow in vancomycin-free medium. Presumably, strains displaying a S-shape growth under vancomycin treatment may be those that are resistant to vancomycin (i.e. exhibit an elevated parental MIC to vancomycin). Can the authors check and discuss whether this is the case? In other words, do the vancomycin MIC of parental strains explain difference in growth between conditions?	

**8**	Lines to 127 to 138. There is quite a large scientific literature on the genetic basis of vancomycin non-susceptibility in S. aureus. Even though the phenotype measured here (growth differences in vancomycin vs. vancomycin-free medium) is different from that commonly measured in other studies (vancomycin susceptibility as measured by minimum inhibitory concentrations at a single time point), it would be relevant to compare if there is any overlap in the genes described in the literature contributing to vancomycin resistance, and the QTL identified here. Unfortunately, finding that "candidate genes that determine a variety of cellular and molecular processes" is not enough to suggest "the biological relevance of functional mapping."	
**9**	Line 153. "Detection of network communities" I am more familiar with the term "module" than "community" to refer to groups of genes involved in the same biological function (e.g. metabolic modules). The title of this section could be re-named as "Detection of network modules".	
**10**	Lines 153 – 178. In order to assess the biological relevance of these 14 modules, and sub-modules described later on, the authors must annotate the biological function of genes within each module and sub-module (such as KEGG terms, metabolic function, etc.). Also, further details on each module like their size (number of genes) and enriched biological function is needed. This could be presented as a supplementary table.	
**11**	Line 176. Check for typo in sentence: "communities, especially M7, M12, and M12, display a similar pattern of time-varying genetic effects." as M12 is repeated twice.	
**12**	Lines 212-259. Biological interpretation of findings is lacking in these paragraphs. The gene name, function and amino acid change of SNPs, QTLs and sub-modules presented here would allow reviewers and readers to assess the biological plausibility of the relationships (inhibition and promotion) described between them.	
**13**	**Comments on impact:**<br>I think major revisions are still needed to judge what Nature journal this paper would be most suitable for.	

**Reviewer #2**<br>*Report received: 23 February 2021*	
**Reviewer #2**	This reviewer has not chosen to waive anonymity. The reviewer's identity can only be shared with representatives of an established journal editorial office.
**Reviewer #2 expertise**<br>*Summarised by the editor*	This reviewer has specific expertise in phenotypic plasticity.
**Editor's comments about this review**	While *Communications Biology* and *Nature Communications* would not require the code to be provided as an executable package, this reviewer offers several suggestions on how to make the omnigenic model more user-friendly and of interest to a broader readership.
**Reviewer #2 comments**	
**Overview**	Summary: This manuscript proposes a novel method of disentangling gene regulatory networks from time series genetic mapping (QTL/GWAS) data. It is applied and discussed with respect to understanding the developmental and genetic bases of phenotypic plasticity and is presented via analysis of bacterial resistance to vancomycin. The method proposed seems to better capture gene interactions than current approaches to analyzing mapping data.<br><br>I was very excited reading this paper and immediately began thinking about how this method could impact my own work in the future. I cannot really speak to the validity or appropriateness of the computations and calculations underlying the method, but the big picture and example presented in the manuscript suggest to me that this will be a powerful tool for disentangling the bases of complex phenotypes. I especially can see it influencing the way evolutionary biologists approach developmental questions.

Specific comments

#	Reviewer comment	Editorial comment
1	**Comment on impact:**<br><br>I think this novel method/framework will influence how the genetic bases of complex traits is investigated if the authors can make their methods user-friendly and accessible.	

**Comments on: Strength of the claims**<br><br>I am convinced that this work could be generally useful. However, I do have three major comments and various minor comments that I think would improve the manuscript, make it more transparent, and more user-friendly (especially for this approach to be widely adopted).		
**2**	First, it is not clear to me why the authors focus so heavily on phenotypic plasticity. It seems to me that this approach should be applicable to any trait for which there is time-series data. Relatedly, it wasn't even clear to me how the authors calculated plasticity in this study (i.e., how were specific values for specific timepoints and strains determined?) While I certainly think a plasticity context is interesting (I am biased in this regard), I think the manuscript could be strengthened if the authors broaden their scope to simply encompass development of any trait and, perhaps, briefly discuss plasticity. Maybe I missed something special about this approach that makes it plasticity-centric.	
**3**	Second, the authors should definitely include a table that describes the necessary inputs for this approach. It took me a while to realize/comprehend that time course data was needed. Such data is not always available—especially for mapping—in many study systems. Therefore, I think a paragraph or two needs to be added to the discussion that address the potential limitations of this method. For example, it may not work in the case of polyphenisms where bimodal or binary phenotypic differences are not observed until adulthood (i.e., in cases where longitudinal data are especially hard to acquire and that have limited windows for variation to be detected).	**Please note that a revision for *Communications Biology* or *Nature Communications* should clearly discuss any limitations of the approach, and would benefit from the requested input table.**

**4**	Third, the hands-on side of the manuscript should be bolstered if the authors desire to have this tool used. Certainly, the description of the approach and its application to the empirical data they use is great, but I could not envision how to actually use this method for my own work. I know that data and code would presumably be available upon acceptance for publication, but the authors should consider how they plan (if at all) to make this method readily implementable by fellow researchers. Doing so would greatly strengthen the impact of this paper and bring about the transformative change the authors describe. As it presently stands, this approach brings together various ways of thinking into a novel conceptual approach, but not a novel practical approach. I sincerely hope it becomes the latter as well.	**Please make code immediately available for reviewer input (ex. GitHub), though *Communications Biology* and *Nature Communications* would not require the code to be formatted into an executable package.**
**Minor comments**		
**5**	At various places in the manuscript the font color changes unexpectedly from black to a more grayish color. Presumably, this is the result of editing or copying from other drafts/communications.	
**6**	L129-130 Please related this description of developmental plasticity more explicitly to the preceding sentence. Related to part of my point 1 above, plasticity was simply the difference between control and stress and then this was taken at various timepoints in development, correct?	
**7**	L131 Please define or describe what composite functional mapping is and if it is unique to your framework	

**8**	L172 Change "A total of" to "The"<br><br>L173 and 'than' after the word 'rather;<br><br>L176 M12 is listed twice<br><br>L189 How were these 45 links identified as actually occurring<br><br>L191 Is it SNPs or modules at this point?<br><br>L196 change 'that trigger' to 'have'<br><br>L197-198 This is a cool level of detail to be able to get to!<br><br>L223-224 This predictive and hypothesis-driving potential could be very useful<br><br>L242 'receive more incoming links that send outgoing links'<br><br>L256 remove 'we actually have'<br><br>L266 remove 'how'<br><br>L276 'leadership'<br><br>L411 italicize *S. aureus*<br><br>L416 italicize *S. aureus*<br><br>L449 remove 'but'<br><br>L459-460 Remove 'published...University' and just include the citations	
**9**	Figure 1A rightmost panel: I am not sure what to make of this figure. Is it meant to show how plasticity was calculated? If so, I am still confused and perhaps more description is warranted.	

10	**Methods**. I know the journal dictates manuscript formatting, but I think this manuscript could greatly benefit from having the methods before the results or co-mingled. This is especially important because of the tool-like nature of this manuscript. Making it easier to see how what was done relates to what was found would be very useful for this manuscript.	**Per *Communications Biology* and *Nature Communications* journal policy, the Methods section should still be placed after the Discussion in a manuscript.**
11	**Reproducibility.** As indicated elsewhere in my review, I think the methods could be made more user-friendly and hands-on so that those not deeply involved in the concepts/theory could implement the approach.	

Reviewer #3

Report received: 24 February 2021

**Reviewer #3**	This reviewer has not chosen to waive anonymity. The reviewer's identity can only be shared with representatives of an established journal editorial office.
**Reviewer #3 expertise** *Summarised by the editor*	This reviewer has expertise in developmental plasticity and evolutionary biology.
**Editor's comments about this review**	This reviewer offers several useful remarks about how to clarify the current omnigenic model.
**Reviewer #3 comments**	
**Overview**	Review of Inferring multilayer interactome networks shaping phenotypic plasticity and evolution by Yang, Jin, He, and Wu<br>Main comments<br>Yang et al. attempt to deduce networks of interactions between loci underpinning a plastic trait. Their goal is laudable and ambitious, and the experimental aspect of their work is fascinating on its own. However, this manuscript, in its current form, suffers from a number of shortcomings and, in my view, requires extensive revisions before it reaches a publishable unit. Below, I highlight some of the major shortcomings of the manuscript in its current form.

Specific comments		
**#**	**Reviewer comment**	**Editorial comment**
**1**	First, the premise of the experiment is not well-justified. Specifically, how does one disentangle the plastic response from the genetic response, given the fact that GWAS, by definition, is linking phenotypic variance directly to the genetic variation? In the absence of such justifications, statements such as "... tremendous inter-strain variation in the pattern … implies the existence of genes that govern phenotypic plasticity." (lines 123-134) are not warranted.	
**2**	Second, the computational model to infer the so-called omnigenic interactome networks, which is the crux of the manuscript, is explained in vague terms. In the introduction section (lines 81 - 94), evolutionary game theory and network theory are invoked, without even the most cursory explanation as to why these theories were utilized to infer interactions from QTLs deduced from a GWAS study. The method section does very little to illustrate how the model works. Statements such as "according to network theory" (line 775) are extremely confusing as they imply that the network theory is something akin to the second law of thermodynamic, while in fact, the sentence is as much useful as a sentence that starts with "according to the theory of evolution …". More precise language and more extensive explanations of the model are required.	**It is essential to carefully revise the language used in the manuscript to ensure that the logic of the approach is clear in response to this comment and those below.**
**3**	Third, the section title "Detection of network communities" (lines 153 - 177), while being crucial for understanding the manuscript, lacks the necessary explanations and elaboration. The developmental modularity theory is introduced without any explanations. (as side note: I presume that BIC (line 166) stands for Bayesian information criterion, but acronyms should always be introduced properly.)	

**4**	Finally, it is not at all clear how measuring genetic effects over 50 hours is a justifiable method to deduce the gene regulatory network that underlies a phenotype when the population in question has undergone roughly 100 generations already (assuming generation time of 30 min). How does adaptive evolution in the presence of antibiotics affect the topology of the interactome networks inferred from the QTLs? In the absence of such justifications, the biological nature and significance of the inferred interactions, such as the supposed “antagonistic” relationship between QTL2728755 and NP2748895, remains elusive. The extensive discussion about the nature of interactions between QTLs and SNPs, as promoters or suppressors, is similarly affected.	**The potential confound of adaptive evolution should be addressed in the Discussion.**
**5**	Aside from the aforementioned comments, given the complexity of the model proposed in this manuscript, it's a pity that its poor structure prevents an adequate comprehension and a fair assessment of its central claim.	**A revision for *Communications Biology* or *Nature Communications* should improve the structure and clarity of the manuscript so that the study can be fairly assessed by reviewers.**
**Minor comments**		
**6**	Line 70: Suggest citing some of the famous papers on the definition of epistasis, including Patrick Philips’ The language of gene interaction (GENETICS July 1, 1998 vol. 149 no. 3 1167-1171).	
**7**	Line 71: “statistical approaches used in the literature cannot reveal the casualty of epistasis and the sigan casualty” => causality not casualty.<br>“..., consistently with a larger amount of growth …” (Line 116) is a slightly misleading characterization of the growth curves in Figure 1, as most strains reach similar levels of abundance under stress compared to the control.	
**8**	Ref 43 should be Maynard Smith, J. and not Smith, J. M.	

**9**	In " … implying their neglected role …" (144) => probably "negligible" and not "neglected".<br><br>Line 682: "differences in equation (1)" => "difference equation (1)"<br><br>Line 752: "a hybrid of the EM algorithm" => a hybrid of the expectation–maximization (EM) algorithm"	
**10**	Line 455: It is either "a wealth of literature" or "a body of literature", but not both.	

**Reviewer #4**<br>*Report received: 23 February 2021*	
**Reviewer #4**	This reviewer has not chosen to waive anonymity. The reviewer's identity can only be shared with representatives of an established journal editorial office.
**Reviewer #4 expertise**<br>*Summarised by the editor*	This reviewer has expertise in biostatistics, population genomics, bacterial GWAS, and evolutionary biology.
**Editor's comments about this review**	This reviewer offers several useful remarks about how to benchmark the current omnigenic model (particularly in comments #2, 5, 11-12), though comments regarding validation in a separate data set (or generation of targeted mutants) would not be necessary for publication in *Communications Biology*.<br><br>For further consideration by *Nature Communications*, demonstration of broader utility is required. The referee provides useful suggestions in points 1, 8, 16 and 17, and at least one of these (or some other equivalent additional dataset) would need to be analysed to demonstrate broader appeal.
**Reviewer #4 comments**	
**Overview**	In this paper, the authors analyse the genetic basis of S. aureus resistance to vancomycin by using a statistical approach based on the omnigenic model. They measured growth curves of 99 strains with and without this stress, and used a

	genome-wide association method for time series data to find significant core SNPs controlling this response. The authors then applied evolutionary game theory to construct an interacting network of core SNPs which can explain the measured response curves. Whereas a 'typical' genetic model of this phenotype would consist solely of the five independently significant QTLs discovered in the first phase; in this model, insignificant SNPs typically have large individual effects on the phenotype, but most of these cancel out due to regulatory effects from other core SNPs in their network. These interactions can be measured and, in some cases, interpreted. The authors imply that many of the regulatory SNPs would be common actors in a range of phenotypes, though this is not tested.<br><br>The omnigenic theory of trait inheritance is an interesting one, worthy of further study in species other than humans. I believe that the statistical approaches and experiments presented here are broadly appropriate to address this question in a limited way. However, with current manuscript I had the following issues with its presentation:

Specific comments

#	Reviewer comment	Editorial comment
1	Broad, sweeping claims are made about the approach being a 'paradigm-shifting' framework. However, only a single phenotype, in a single species, in a specific strain collection was tested. A key feature of the omnigenic model is that the regulatory SNPs regulate many phenotypes, but as the authors only test one phenotype they cannot identify whether the links in their regulatory networks are shared.	**This point would only have to be mentioned as a limitation for *Communications Biology*.**<br><br>**Please refer to the comments above for requirements for *Nature Communications.***
2	The omnigenic model is essentially taken as a prior for the analysis here. All SNPs are included from the bottom-up, as a feature. But what about other models? Does this model the data better than say a model with the QTLs? Or a polygenic model such as GCTA and LDAK? Without these comparisons, I do not think that the authors can claim that their model and approach is superior in terms of predictive power or mechanistic understanding of these traits.	***Communications Biology* and *Nature Communications* would require any revision to benchmark the omnigenic model to polygenic analyses.**

**3**	There is not enough clarity, particularly in the introduction and conclusion, on what it actually is that the authors are testing. There are a lot of buzzwords, but from my reading the main thing this manuscript does show us one way to construct an omnigenic model for a trait, and compare it with some of the QTLs found in a GWAS. I would encourage the authors to spend more time introducing monogenic, polygenic, omnigenic models, and missing heritability. Give the biological/genetic interpretations of each, and compare and contrast them. Additionally, note the difference between phenotype prediction and understanding the biological basis of phenotypes – two related but different goals of these models.	**A revision for either *Communications Biology* or *Nature Communications* would benefit from discussing the broader context of omnigenic theory vs. polygenic models.**
**4**	There is a long part of the paper and the figures showing and describing links in networks. Without either strong evidence that this model is superior, or biological interpretation (SNPs in this section are simply numbered), this is both speculative and offers poor explanatory power.	
**5**	The section on 'model validation' was not particularly convincing. It mostly seemed to repeat the same experiment, as the essentially the same phenotype and analysis were redone. This felt like a bit of a missed opportunity: if the authors had made mutants which can directly measure effects of SNPs, they could have tested regulatory predictions from the constructed networks, and compared these with a model of individual QTLs. At least, comparing predictive power of the network approach versus the QTL approach in a new dataset would have been more valuable.	**At a minimum for *Communications Biology*, any revision should compare the predictive power of the omnigenic model vs. a more standard QTL approach using the current data set. However, only *Nature Communications* would require validation in a new dataset.**
**Comments on: Impact**		
**6**	I do think that this paper could be an interesting addition to the microbial statistical genetics literature. As far as I am aware, no one has previously explored the omnigenic model in bacteria before. But to do so effectively, the authors need to be less bold in their claims, clearly compare the genetic models they are testing, and be refocus to target a comparison between omnigenic and polygenic models specifically.	

**7**	Overall, I can see the rationale for this model, but is based on asserting that all SNPs have an effect: can we connect these in a way to get the pattern in this (single) GWAS? I don't see evidence here that this model refutes just the significant QTLs, or a standard polygenic model, predicting the data equally well.	
**8**	For a more impactful paper which would inspire others to use this model, I would expect to see more targeted experiments, using new strains or mutants, which tested these models predictive and explanatory power. I would also expect to see more reusable code, which include some continuous integration testing.	**Again, experimental validation in new strains or mutants would not be necessary for *Communications Biology*, though the code should be made available.**
**Comments on: Strength of the claims**		
**Required:**		
**9**	The effects of population structure are dealt with only in passing, but are a major issue for many GWAS studies. I agree that the QQ plot and Manhattan plot look broadly correct, but would need to also see:<br>a. A phylogenetic tree of the isolates, ideally with some summary of the phenotype mapped on as a trait.<br>b. More information of how the eight subpopulations were identified and removed.<br>c. A comparison of a standard linear mixed model versus a quantitative summary statistic of the trait (for example the growth rate and/or carrying capacity)	
**10**	More biological inference of the *S. aureus* results integrated into the main text, moving table S1 into the main results, and describing SNPs in terms of known function rather than just position. In particular, a biological interpretation of the modules of SNPs would make the model more convincing.	

11	Make a clear comparison between the omnigenic model of trait heritability, and a mono/polygenic model. Show which has better explanatory power. I would note that more sub-significant SNPs affecting a phenotype is well established and modelled by e.g. the GCTA model. Implicitly, the GCTA model assumes that all SNPs have small effects. So the difference with the omnigenic model is that many have large effects, but they cancel out through interactions. But, which is the model better supported by the data here?	**This point would be necessary for both *Communications Biology* and *Nature Communications*.**
12	In missing heritability, calculating h^2, and h^2 from the QTLs would be a really helpful way to compare these models. Adding the significant SNPs from the module approach to a h^2 calculation would also be informative.	**This point would be necessary for both *Communications Biology* and *Nature Communications*.**
13	Likewise, using a method such as SpydrPick or just simple associations of interaction terms to find epistasis via 'conventional' means would be useful to compare to interactions in the networks (even if the result is that this is underpowered).	
14	The cancelling out of strong effects through interactions in the omnigenic model is necessary to reconcile individual effects at the lower level with no effect overall (the QTLs in the GWAS). What would the alternative look like? Is there a way to not have an omnigenic model and still get the GWAS effects?	
**Optional:**		
15	The authors only examine SNPs in the core genome, but the accessory genome is known to have an important role in adaptive response in bacteria (e.g. http://dx.doi.org/10.1038/s41559-017-0337-x, and many others). Could the accessory genome variation be included in an omnigenic model?	
16	If the same regulatory loci and interactions were found to hold for other phenotypes in the species, the model and approach here would be more convincing. A multi-phenotype study, perhaps RNA-seq, would be most powerful of all. There are some public datasets available with this kind of data.	**This point would not be necessary for *Communications Biology*.**

**17**	Validation of the model by creating mutants based on the networks speculated above, to test whether these SNPs do affect regulation would add a lot. For example, can both the network approach, and QTL approach be used to predict phenotype response in a recombinant mutant. Where these predictions are divergent, then testing which phenotype the mutant has would be a powerful confirmation of that model.	**This point would not be necessary for *Communications Biology*.**
**Additional minor comments:**		
**18**	In the introduction: omnigenic theory is describe as all traits being controlled by all genes. My understanding is that there are common regulatory genes which are involved in many phenotypes, not that every gene is functional. How could the authors reconcile their assertion that all genes are involved with the frequent loss of genes in bacteria?	
**19**	Network is used many times with a specific definition in the introduction.	**The reviewer may mean "without" rather than "with" here.**
**20**	'millions of millons of gene pairs' – in a typical bacterial population there would be around 10^7 genes. Be more specific if possible. Also, this is consistently stated to be computationally intractable. Networks with this many edges are possible to analysed however.	
**21**	Inferring this many interactions from a few samples is still likely to be underpowered, even with improved analysis (even though the time series does add power). How confident are the authors that their network is the only correct one?	
**22**	'Statistical approaches cannot infer the casuality of epistasis'. Is this specific to epistasis? Approaches such as Mendelian randomization, or that of Pearl et al have attempted this I believe. Also a typo in 'causality'.	
**23**	'design an efficient and effective gene editing program'. In bacteria? More broadly?	

**24**	A deficiency of linear additive models is stated as missing random effects, but linear mixed models, the most commonly used models in GWAS and breeding, do include random effect terms.	
**25**	Is bDSW a standard acronym? I found its use confusing, and would suggest that simply 'edges' or 'interactions' would be easier to follow.	
**26**	I noted that the genetic effect vs time curves mostly show effect during the growth phase (rather than stationary). Is this because this is where phenotype difference/derivative with respect to time is greatest, and there is greatest power?	
**27**	'find that their effect curves are quite flat, implying their neglected role in shaping phenotypic plasticity.' How does this implication follow?	
**28**	I appreciate that minimizing BIC is a good quantitative way to choose the number of modules, but it might be informative to use a lower number, and see if any obvious biological pattern is apparent (as is, many of the module effects look very similar).	
**29**	The beginning of the section 'multilayer genetic networks' could more clearly and succinctly be described as 'we recursively subdivide communities'.	
**30**	On sparsity: is finding a sparse network a necessary property the method of subdivision, or a finding when applied to this dataset?	
**31**	'Most links are directional rather than reciprocal' – invoking interactions between populations of animals is unlikely to be universal, does it really apply to SNPs and antimicrobial resistance?	
**32**	The discussion restates a lot of the results, and could be trimmed. Some of the biological interpretation should be in the results.	

**33**	'Our model integrates the theories of two recent perspective articles published in Cell by J. Pritchard and M. Snyder both at Stanford University'. The citations will suffice.	
**Comments on: Reproducibility**		
**34**	The code URL isn't listed in the manuscript, but I found it (with a typo) in the reporting summary. In such a manuscript, the code needs to be more prominent, and in a state where others can use it. At the moment it is fairly unstructured, has local links to files, and could certainly not be reused or replicated by others.	
**35**	Analysis in this paper was written from scratch, no existing software was used. How can we be sure that no errors occurred in this process? The final model is complex. Some testing of the code itself is needed.	**As stated earlier: code should be made available for reviewers.**

Open Research Evaluation	
**Data Availability**	
**Data Availability Statement**	The Nature Portfolio journals strongly support public availability of data and custom code associated with the paper in a persistent repository where they can be freely and enduringly accessed or as a supplementary data file when no appropriate repository is available. If data and code can only be shared on request, please explain why in your data Availability Statement, and also in the correspondence with your editor. For more information, please refer to https://www.nature.com/nature-research/editorial-policies/reporting-standards#availability-of-data<br><br>Please ensure that datasets deposited in public repositories are now publicly accessible, and that accession codes or DOI are provided in the "Data Availability" section. As long as these datasets are not public, we cannot proceed with the acceptance of your paper. For data that have been obtained from publicly available sources, please provide a URL and the specific data product name in the data availability statement. Data with a DOI should be further cited in the methods reference section<br><br>See our policy page for more details.
**Mandatory data deposition**	http://www.nature.com/authors/policies/availability.html#data<br><br>**Please note that whole-genome sequencing data and GWAS summary statistics are both mandatory data types that must be deposited in a public database for publication in any Nature Portfolio journal.**
**Recommended data deposition**	Nature Portfolio policies include a strong preference for research data to be archived in public repositories. For data types without specific repositories, such as growth plasticity, we recommend that data are deposited in a generalist repository such as figshare or Dryad. If you need help

	complying with this policy, or need help depositing and curating your research data (including raw and processed data, text, video, audio and images) you should consider:<br>● Contacting Springer Nature's Research Data Helpdesk for advice,<br>● finding a suitable data repository for your data,<br>● Or uploading your data to Springer Nature's Research Data Support service.<br><br>Please note there are fees for using Springer Nature's Research Data Support service.
**Source data**	The following figure panels should be accompanied by the underlying source data: **Figure 1a, 2a/c, 4-6**<br><br>Source data files will be mandatory prior to publication in a Nature Portfolio journal.
**Data citation**	Where datasets are hosted in public repositories that provide datasets with Digital Object Identifiers (DOIs), we encourage these datasets to be formally cited in reference lists. Citations of datasets, when they appear in the reference list, should include the minimum information recommended by DataCite and follow journal style. For example:<br>Hao, Z., AghaKouchak, A., Nakhjiri, N., Farahmand, A. Global Integrated Drought Monitoring and Prediction System (GIDMaPS) Data sets. *figshare*. http://dx.doi.org/10.6084/m9.figshare.853801 (2014)<br><br>Citing and referencing data in publications supports reproducible research, by increasing the transparency and provenance tracking of data generated or analysed during research. Citing data formally in reference lists also helps facilitate the tracking of data reuse and may help assign credit for individuals' contributions to research. A number of Springer Nature imprints are signatories of the Joint Declaration on Data Citation Principles, which stress the importance of data resources in scientific communication.
**Data Publication Recommended**	We suggest that you consider publishing your omnigenic model as a Data Descriptor in *Scientific Data*. See the journal

	website for details.<br>https://www.nature.com/sdata/
**Code Availability**	
**Code Availability**	Please provide a separate statement regarding code availability, and a functional GitHub web-link to access the source code. This should be provided both in the Reporting Summary as well as in the manuscript.<br><br>See our policy page for details.
**Code Citation**	In addition to making the custom code available, we ask that you ensure that the version of the code/software described in the paper is deposited in a DOI-minting repository (eg, Zenodo) and that this DOI is also cited in the main Reference list. See here for details.
**Methods assessment and reproducibility**	
**Statistical Evaluation**	• Wherever statistics have been derived (e.g. error bars, box plots, statistical significance) the legend needs to provide and define the n number (i.e. the sample size used to derive statistics) as a precise value (not a range), using the wording "n=X biologically independent samples/animals/cells/independent experiments/n= X cells examined over Y independent experiments" etc. as applicable.<br><br>• We strongly discourage deriving statistics from technical replicates, unless there is a clear scientific justification for why providing this information is important. Conflating technical and biological variability, e.g., by pooling technically replicates samples across independent experiments is strongly discouraged. (For examples of expected description of statistics in figure legends, please see the following https://www.nature.com/articles/s41467-019-11636-5 or https://www.nature.com/articles/s41467-019-11510-4).

**Figure legends**	• The figure legends must indicate the statistical test used. Where appropriate, please indicate in the figure legends whether the statistical tests were one-sided or two-sided and whether adjustments were made for multiple comparisons.<br>• For null hypothesis testing, please indicate the test statistic (e.g. F, t, r) with confidence intervals, effect sizes, degrees of freedom and P values noted.<br>• Please provide the test results (e.g. P values) as exact values whenever possible and with confidence intervals noted.<br>• **Please note that the information on whether the statistical test used was one-sided or two-sided, where appropriate, is missing in the legend of figure 1b.**
**Reproducibility**	Please state in the legends how many times each experiment was repeated independently with similar results. This is needed for all experiments, but is particularly important wherever results from representative experiments (such as micrographs) are shown. If space in the legends is limiting, this information can be included in a section titled "Statistics and Reproducibility" in the methods section.
**Methods Descriptions**	The Methods must contain sufficient detail such that the work could be repeated. It is preferable that all key methods be included in the main manuscript, rather than in the Supplementary Information.
**Data presentation**	Please ensure that data presented in a plot, chart or other visual representation format shows data distribution clearly (e.g. dot plots, box-and-whisker plots). When using bar charts, please overlay the corresponding data points (as dot plots) whenever possible and always for n ≤ 10. (Please see the following editorial for the rationale behind this request and an example https://www.nature.com/articles/s41551-017-0079).
